# Efficacy Evaluation of Plant Products in the Treatment of Erectile Dysfunction Related to Diabetes

**DOI:** 10.3390/nu13124520

**Published:** 2021-12-17

**Authors:** Stefania Nobili, Elena Lucarini, Stefania Murzilli, Arianna Vanelli, Lorenzo Di Cesare Mannelli, Carla Ghelardini

**Affiliations:** 1 Department of Neurosciences, Imaging and Clinical Sciences, “G. d’Annunzio” University of Chieti-Pescara, Via L. Polacchi 11, 66100 Chieti, Italy; 2 Department of Neuroscience, Psychology, Drug Research and Child Health-Neurofarba-Section of Pharmacology and Toxicology, University of Florence, Viale Pieraccini 6, 50139 Florence, Italy; elena.lucarini@unifi.it (E.L.); lorenzo.mannelli@unifi.it (L.D.C.M.); carla.ghelardini@unifi.it (C.G.); 3 Nutrilinea S.R.L., Via Gran Bretagna 1, 21031 Gallarate, Italy; stefania.murzilli@nutrilineasrl.it (S.M.); arianna.vanelli@nutrilineasrl.it (A.V.)

**Keywords:** erectile dysfunction, diabetes, *Panax ginseng*, *Moringa oleifera*, rutin, animal studies

## Abstract

Erectile dysfunction affects more than 50% of diabetic male patients, with a higher prevalence compared with the general population. Age, clinical factors, and lifestyle habits have been suggested to contribute to the pathophysiology and worsening of erectile dysfunction in diabetic patients. First- and second-line standard treatments are represented by phosphodiesterase type 5 (PDE5) inhibitors and alprostadil, respectively. However, natural compounds have been suggested to ameliorate this clinical condition. This study aims to preclinically characterize the potential synergism among plant-derived products for the improvement of erectile dysfunction in the diabetic condition. The effects of a nutritional supplement composed of *Panax ginseng*, *Moringa oleifera* and rutin, as single agents or as a mixture, were evaluated in a streptozotocin (STZ)-induced diabetic rat model with erectile dysfunction. The treatment efficacy was evaluated by measuring sexual-related parameters (i.e., mount and intromission latencies, the mount and intromission frequencies and the ejaculation latency). Results showed that only the mixture was able to significantly reduce the diabetes-related delay in mount latency (*p* < 0.01). Substantial similar effects were observed by measuring the intromission latency and the mean number of mounts was very similar between rats treated with the mixture and controls. Single agent treatments showed very low effects in terms of intromission frequency, whereas the mixture was able to increase this parameter. Additionally, a statistically significant reduced ejaculation latency was observed in rats treated with the mixture compared with the STZ control. These results are in agreement with the available literature and suggest that the study mixture may ameliorate sexual behavior compared with the administration of the study natural compounds as single agents in diabetic rats. Further preclinical and clinical studies are needed to perform a more comprehensive evaluation of the efficacy and safety of the study mixture.

## 1. Introduction

Recent data estimate that the global diabetes prevalence in 2019 was 9.3%, and a progressive increase is expected in the next 10 and 25 years [1]. Normal sexual function may be impaired in both male and female diabetic patients and erectile dysfunction is one of the most common diabetes-related sexual dysfunctions in men [2]. Overall, erectile dysfunction affects more than 50% of diabetic male patients, with a higher prevalence compared with the general population (i.e., odds ratio about 3.5) [3]. In particular, age and duration of diabetes have been associated with a higher prevalence of erectile dysfunction in type 2 diabetic patients [4]. Overall, several clinical factors, mainly related to vascular, neurological and hormonal conditions, have been suggested to contribute to the pathophysiology of erectile dysfunction in these types of patients [2]. In particular, the impairment of corpus cavernosum smooth muscle relaxation due to the endothelial-derived nitric oxide (EDNO) induced by end products of glycosylation [5,6], diabetic neuropathy [7], low levels of testosterone [8], and obesity [9] may play a role in this condition.

In addition, lifestyle factors related, for instance, to diet, smoking, sedentary habits, alcohol and drug (e.g., cocaine, opioids) abuse, which are well recognized risk factors of erectile dysfunction [10], may also worsen this pathological condition in diabetic patients [11].

Pharmacological strategies to treat erectile dysfunction related to diabetes do not differ from those used in the diabetes unrelated form and include oral highly selective phosphodiesterase type 5 (PDE5) inhibitors (e.g., sildenafil, tadalafil) as first-line treatment and prostaglandin E1 alprostadil as second-line treatment. Penile erection is physiologically mediated by a nitric oxide (NO)-cyclic guanosine monophosphate (cGMP) pathway. PDE5 is a regulator of vascular smooth muscle contraction through the regulation of cGMP [12]. In the erectile dysfunction condition, the pharmacological inhibition of PDE5 enhances relaxation of smooth muscle by NO, which activates cGMP, increasing its level in the corpum cavernosum, thus stimulating penile erection [13]. Interestingly, the positive effect of tadalafil in ameliorating high fat diet-induced erectile dysfunction was observed in a non-genomic rabbit model of metabolic syndrome [14]. Metabolism has also been associated with other relevant findings in the same animal model, such as the reduction of the mRNA expression levels of inflammatory, profibrotic and hypoxia biomarkers [15,16]. Alprostadil, by stimulating the intracellular adenylate cyclase pathway, promotes the synthesis of cyclic adenosine monophosphate (cAMP), which results in a corresponding decline in cytosolic calcium. This occurrence relaxes the cavernosal smooth muscle, leading to penile erection [17].

Despite the wide use of PDE5 inhibitors among men with erectile dysfunction, a high percentage of dropout occurs mainly due to lack of efficacy (>20%) or side effects (>10%) [18]. Alprostadil is administered by intracavernosal injection or by urethral suppository, in both cases requiring patient training and producing discomfort [19].

Thus, for these and other practical or personal reasons, there is a wide use of nutraceuticals and dietary supplements among men with erectile dysfunction. Natural compounds included in these formulations may contribute to the mitigation and treatment of this condition [20]. However, most plant compounds considered endowed by positive effects in the treatment of erectile dysfunction have been investigated only in vitro and/or in vivo models. Few plant compounds (e.g., *Crocus sativus* L. [21], *Tribulus terrestris* [22], *Panax ginseng* [23,24,25] have been tested in humans affected by this syndrome. Among these, the most investigated is *P. ginseng*, which has been evaluated either as a single agent [23,24] or in combination with *M. oleifera* and rutin [25] in clinical trials with promising results.

Thus, this study aims to preclinically characterize the potential synergism among plant-derived products for the improvement of erectile dysfunction in the diabetic condition. In particular, the effects of a nutritional supplement composed of *Panax ginseng* (*P. ginseng*, Araliaceae family), *Moringa oleifera* (*M. oleifera*, Moringaceae family) and rutin, three natural products active also on microcirculation, were evaluated in a streptozotocin (STZ)-induced diabetic rat model with erectile dysfunction. Single agent and combination studies have been performed.

## 2. Results

**Figure 1:**
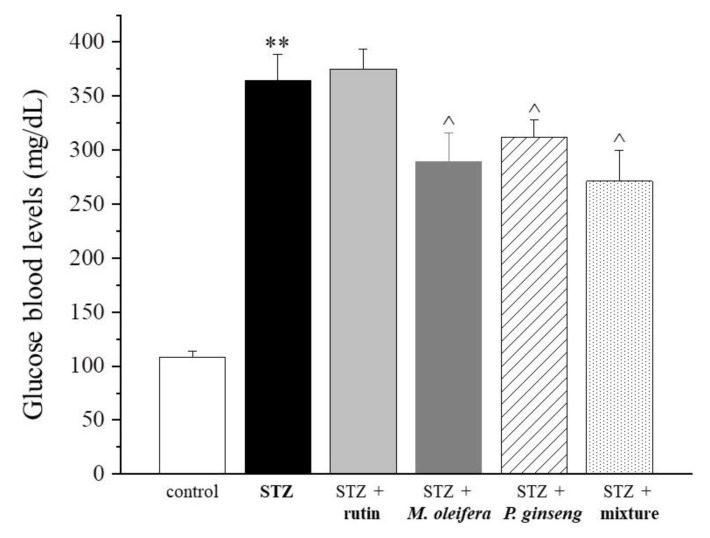
Glucose blood levels. Results are reported as mean ± S.E.M. of 10 rats. ** *p* < 0.01 vs. control; ^ *p* < 0.05 vs. STZ.

After STZ-induced pancreatic damage, higher levels of blood glucose were observed in diabetes rat models compared with the control (364.8 ± 23.5 vs. 108 ± 6.2 mg/dL). As shown in Figure 1 and Table 1, after 4 weeks of the repeated administration of *M. oleifera* and *P. ginseng* extracts administered as single agents, as well as of the complete mixture, blood glucose levels were significantly reduced compared with the STZ control (*p* < 0.05). Rutin per se was ineffective. However, neither single agents nor the mixture reverted to the diabetic model.

**Figure 2:**
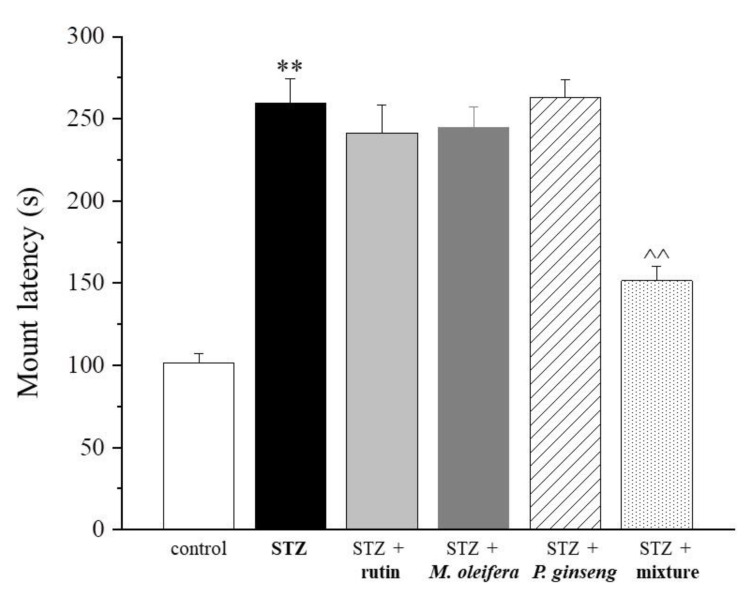
Mount latency. Results are reported as mean ± S.E.M. of 10 rats. ** *p* < 0.01 vs. control; ^^ *p* < 0.01 vs. STZ.

Regarding the sexual-related parameters, a significant improvement by the use of the mixture emerged. In Figure 2 and Table 2, the mount latency analysis revealed that only the mixture was able to significantly reduce the diabetes-related delay (*p* < 0.01). In particular, a reduction of about 2 min and an increase lower than 1 min in the mount latency between rats treated with the mixture and the STZ group or the control group, respectively, were observed.

**Figure 3:**
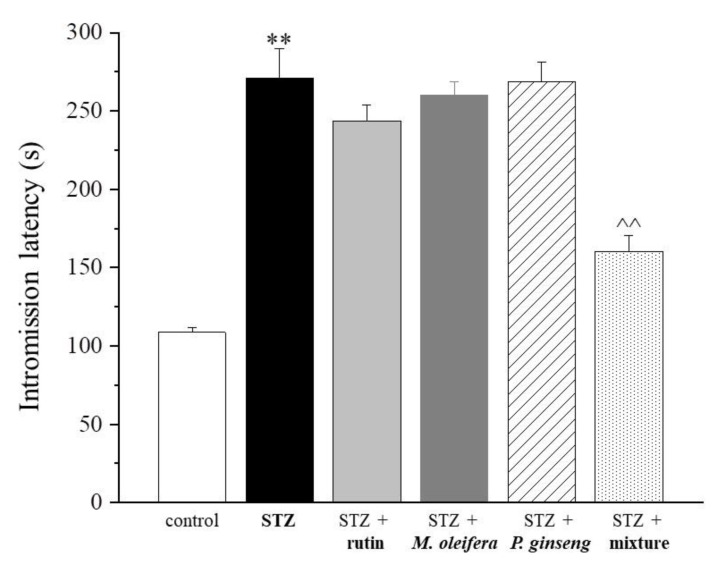
Intromission latency. Results are reported as mean ± S.E.M. of 10 rats. ** *p* < 0.01 vs. control; ^^ *p* < 0.01 vs. STZ.

**Figure 4:**
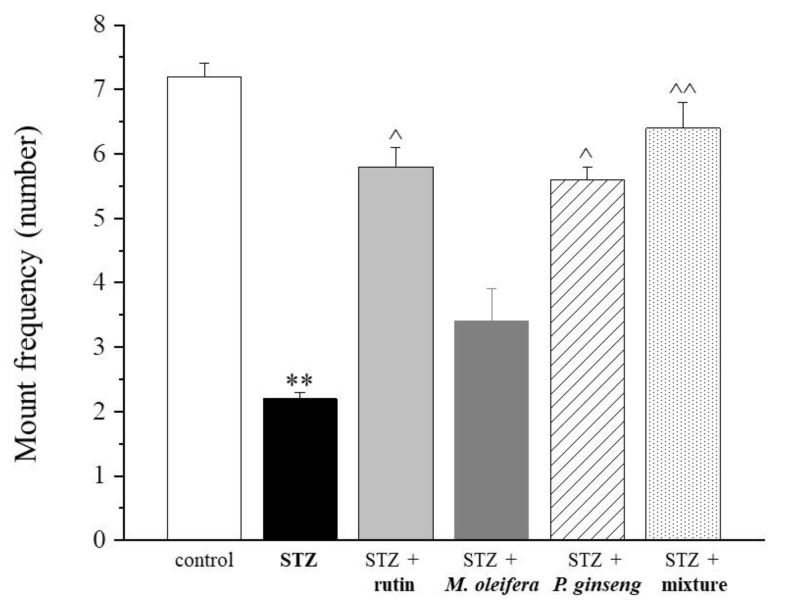
Mount frequency. Results are reported as mean ± S.E.M. of 10 rats. ** *p* < 0.01 vs. control; ^ *p* < 0.05 and ^^ *p* < 0.01 vs. STZ.

Substantial similar effects were observed by measuring the intromission latency (Figure 3 and Table 3). The mount frequency was stimulated by both rutin and *P. ginseng* extracts administered as single agents and to a higher extent by the mixture (Figure 4 and Table 4).

**Figure 5:**
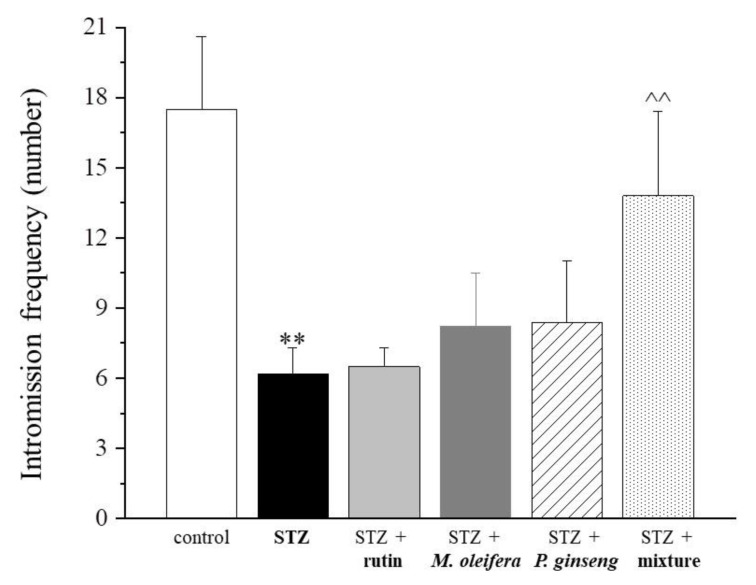
Intromission frequency. Results are reported as mean ± S.E.M. of 10 rats. ** *p* < 0.01 vs. control; ^^ *p* < 0.01 vs. STZ.

**Figure 6:**
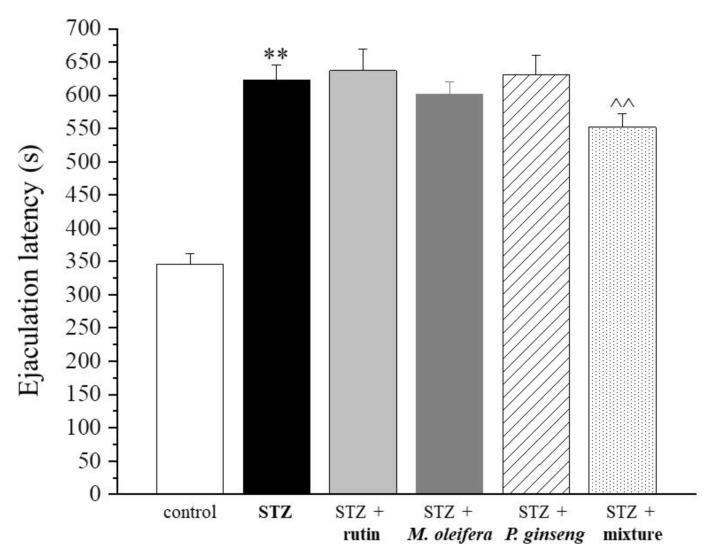
Ejaculation latency. Results are reported as mean ± S.E.M. of 10 rats. ** *p* < 0.01 vs. control; ^^ *p* < 0.01 vs. STZ.

In particular, the mean number of mounts was very similar between rats treated with the mixture and controls. In relation to the intromission frequency parameter, the three single agent treatments showed very low effects (i.e., rutin < *M. oleifera* < *P. ginseng*). The mixture was instead able to increase the frequency of intromission (Figure 5 and Table 5). Finally, a statistically significant reduced ejaculation latency was observed in rats treated with the mixture compared with the STZ control (Figure 6 and Table 6).

## 3. Discussion

Current standard pharmacological strategies to treat erectile dysfunction include PDE5 inhibitors and prostaglandin E1 alprostadil as first- and second-line treatments, respectively [26]. The PDE5 inhibitors are efficacious in a high percentage of patients and randomized clinical trials [27,28] and open label trials [29] have shown the efficacy and substantial good tolerability of first-generation PDE5 inhibitors also in diabetic patients. However, PDE5 inhibitors may be used without medical prescription in real life, and this occurrence may contribute to increase unexpected side effects and discontinuation [30]. Data on the second generation PDE5 inhibitors (e.g., avanafil), synthesized with the aim of reducing problems related to the first generation, do not seem to significantly satisfy the purpose [31].

The use of alprostadil is impaired by the development of side effects, mainly including pain at the site of injection (3–44%) and prolonged erection (1–8%) [32,33].

Medicinal plants have wide use as a remedy for a very large plethora of diseases. Several plant compounds (e.g., alkaloids, terpenoids, steroids and polyphenols) have been suggested to have some activity in erectile dysfunction based on in vitro and or in vivo studies (reviewed in [10]). The use of well-established phytochemicals, if efficacious, could at least in part overcome the side effects and the consequent treatment discontinuation occurring during treatment with PDE5 inhibitors or alprostadil.

The present study showed the efficacy of a mixture composed of *P. ginseng* extract, *M. oleifera* extract and rutin in improving erectile dysfunction in a diabetic rat model according to mounting frequency, intromission frequency and mounting latency experiments. Based on the obtained results, the three products were inactive or only partially active when administered at biologically relevant doses as single agents, whereas their combination was significantly effective when compared with controls, revealing a synergistic effect. To our knowledge, this is the first study that investigated the effects of this mixture on the sexual function in diabetic rats, while a number of studies have evaluated the effects of *P. ginseng* [34,35,36,37] and *M. oleifera* [38,39,40,41] as single agents in the same (diabetic rats) or other (normal rats) settings.

In particular, preclinical studies performed in diabetic male rat models have shown improvement of erectile function by treatment with *P. ginseng* (e.g., Korean Red Ginseng) [34] or its active ingredients, such as Ginsenoside Rg3 from *P. ginseng* C.A. Meyer [35] or *P. notoginseng* saponins [36,37]. From a mechanistic point of view, the antioxidant effect of *P. ginseng* exerted in the corpus cavernosum of rats [34,35], associated with the restoration of Akt activity [36], and to the increased expression of eNOS and of the levels of NO and cGMP [37], have been suggested as the main contributors to the positive effects of *P. ginseng* in erectile dysfunction in diabetic rat models.

*M. oleifera* has long been used in traditional medicine. Many studies have reported its antioxidant, anti-hyperglycaemic, anti-dyslipidaemia activities, tissue-protective (liver, kidneys, heart, testes and lungs), analgesic, antihypertensive and immunomodulatory actions [42].

The leaf extracts of *M. oleifera* Lam contain active compounds, including gallic acid, catechin, chlorogenic acid, epicatechin, rutin, quercetin and kaempferol. A number of in vivo studies have evaluated the association between *M. oleifera* administration and the activity of selected enzymes that play a potential role in erectile dysfunction [38,39,40,41].

Phenolic extracts from the leaves of *M. oleifera* have been shown to inhibit key enzymes associated with erectile dysfunction and oxidative stress in penile tissues of rats [40]. In particular, the phenolic extract from *M. oleifera* leaves, in addition to inhibiting angiotensin-I converting enzyme (ACE) and arginase in vitro activity in a dose-dependent manner, also showed radical (OH∗, NO∗) scavenging properties and Fe^2+^-chelating abilities [40]. *M. oleifera* has also been shown to be a potent inhibitor of soluble epoxide hydrolase (sEH) (IC_50_ 1.7 ± 0.1 µg/mL), an enzyme whose inhibition could improve penile erection [39]. Results of an in vitro/in vivo study showed that *M. oleifera* seed extract increased the sexual function in normal rats, as well as normalized diabetes-induced sexual dysfunction in diabetic rats. In particular, *M. oleifera* seed extract was able to relax phenylephrine pre-contracted isolated corpus cavernosum smooth muscle of rats to a significant major extent compared with controls, although sildenafil was more potent than *M. oleifera* [39]. Additionally, *M. oleifera* extract was able to significantly improve the sexual activity of male diabetic rats by increasing mounting frequency, intromission frequency and ejaculatory latency and by decreasing mounting latency compared with controls [39].

A recent study investigated the effect of a diet including *M. oleifera* leaves and seeds on the activity of candidate enzymes, such as acetylcholinesterase (AChE), monoamine oxidase (MAO), ACE, adenosine deaminase (ADA) and arginase in relation to erectile dysfunction in penile tissues of diabetic male rats treated with or without Acarbose (ACA). Thiobarbituric acid reactive species were also evaluated. The activities of AChE, MAO, ADA, ACE, arginase and thiobarbituric acid reactive species levels were significantly restored in the diabetic rats, independently from the ACA treatment, compared with diabetic controls [41].

A hydroethanolic extract of *M. oleifera* leaves was investigated in male rats under stress conditions obtained by long-term immobilization (i.e., 12 h for 7 days) in order to establish its potential effects on the suppression of monoamine and PDE-5 activities, serum testosterone and corticosterone levels, and histomorphological changes in the testes. Antioxidant and MAO type B (MAO-B) suppression activities were shown, as well as improved sexual performance by decreasing intromission latency and increasing intromission frequency by low doses of the extract. Suppression of PDE-5 activity decreased serum corticosterone levels and increased serum testosterone levels [37].

Rutin is a flavonoid glycoside formed from quercetin linked to the disaccharide rutinose, characterized by antioxidant, antidiabetic and anti-lipid peroxidation actions. Interestingly, plant extracts containing high concentrations of rutin have been shown to inhibit arginase in vitro, increasing the amount of arginine available for conversion into NO by nitric oxide synthetase (NOS), as well as exerting a direct inhibitory effect of PDE5 [43].

In a diabetic-induced erectile dysfunction rat model, increased levels of testosterone and penile cGMP content have been shown [44]. Rutin is able to counteract oxidative stress and endothelial damage that is generated in the course of diabetic disease, resulting in a significant increase in sexual function [44] and in some positive effects on male infertility [45]. At the tissue level, in fact, the signs of inflammation, lipid peroxidation, oxidative stress and cell damage induced by hyperglycemia seem to be significantly reduced by rutin [44,46].

To date, some randomized double-blind, placebo-controlled clinical trials have been carried out in men with mild–moderate erectile dysfunction and showed a slightly improved sexual function in terms of penile response and ejaculation, following the administration of 350 mg standardized Korean ginseng berry extract for 8 weeks compared with the placebo [23]. A further trial, based on the International Index of Erectile Function (IIEF-5) score, evidenced an advantage in terms of efficacy of Korean Red Ginseng (KRG) (1000 mg three times a day for 12 weeks) compared with the placebo. In fact, 66.6% of KRG-treated patients showed improved erection, which is significant in the global efficacy question (*p* < 0.01) [24].

Currently, *M. oleifera* and rutin as single agents have not been tested for erectile dysfunction in humans.

Interestingly, a recent randomized prospective clinical trial evaluated the contribution of the study mixture, i.e., *P. ginseng* extract (ginsenosides 10%), *M. oleifera* extract (saponins 20%) and rutin (95%), in patients affected by erectile dysfunction with organic etiology treated with tadalafil [25]. Patients received tadalafil (5 mg/day) plus the mixture or tadalafil (5 mg/day) plus placebo for 3 months. Interestingly, the patient arm that received the PDE5 inhibitor plus the mixture showed a significantly higher IIEF-5 score compared to the patient arm that received tadalafil plus placebo (*p* < 0.0001). These data highlight the potential of the study mixture in ameliorating erectile dysfunction.

Overall, in the aforementioned clinical studies, including *P. ginseng* as a single agent [23,24] or combined with *M. oleifera* and rutin [25], *P. ginseng* administration was daily and lasted from 2 to 3 months. A recent review [47] reported that, independently from the pathology of patients, when *P. ginseng* was administered for more than 6 months, a higher efficacy was usually shown compared with shorter administrations. Interestingly, no toxic effect was shown when *P. ginseng* was administered for 12 months in a randomized controlled trial including patients with moderate chronic obstructive pulmonary disease [48]. Clinical trials performed in different clinical conditions in which *M. oleifera* has been administered for periods ranging from 40 days to 6 months are also available and no side effects have been reported [49,50,51]. Slightly shorter administration times, i.e., 2 and 4 months, have been planned for two ongoing clinical trials investigating rutin in diabetic [52] and hemodialysis [53] patients, respectively.

However, real-word published data useful to establish the potential most efficacious and safe treatment schedule of the study mixture in erectile dysfunction are not currently available. Thus, to date, it is not possible to establish the adequate duration of the mixture treatment, although 4–6 months could be a reasonable treatment period to be investigated in a controlled clinical trial.

Our study has some limitations. In particular, our explorative study does not provide information useful to explain the mechanisms by which the study mixture contributes to the improvement of the sexual functions in diabetic rats. Determination of PDE-5 activity, as well as the evaluation of the levels of relevant steroid hormones or the investigation of histopathological changes in germinal cells and interstitial tissue of testis, could have allowed a more comprehensive characterization of the effects of the study mixture. However, the obtained results are in agreement with the available literature and suggest that the study mixture may ameliorate sexual behavior compared with the administration of the study natural compounds as single agents in diabetic rats. Further preclinical and clinical studies are needed to perform a more extensive evaluation of the efficacy and safety of the study mixture.

## 4. Materials and Methods

### 4.1. Animals

Sprague-Dawley rats (Envigo, Varese) that weighed about 200–220 g at the start of the experiment were used, housed in the Laboratory Animal Stable Center of the University of Florence (Ce.S.A.L.). The animals were placed in cages of 26 cm × 41 cm in environments with a temperature of 23 ± 1 °C with a 12-h circadian cycle and fed according to the standard diet and ad libitum water. All treatments were carried out following Directives 2010/63/EU of the European Parliament and of the Council of the European Union (22 September 2010) regarding the protection of animals used for scientific purposes. The ethical policy of the University of Florence conforms to the National Institutes of Health Guide for the care and use of laboratory animals (NIH Publication n. 85-23, revised 1996; University of Florence Assurance n. A5278-01). Formal approval for conducting the experiments was given by the university council. The experiments were carried out trying as much as possible to minimize the suffering of the animals and their number.

### 4.2. Drugs and Supplies

*P. ginseng C.A. Mey* hydroalcoholic root extract—10% in ginsenosides (i.e., 50 mg ginsenosides) was obtained by Jiaherb Phytochem (Xi’an, China). *M. oleifera* seed hydroalcoholic extract—20% in saponins (i.e., 20 mg saponins) was obtained by Sergio Fontana s.r.l. (Canosa di Puglia, Italy). Rutin from *Sophora japonica* L. flower extract 95% (i.e., rutin 50 mg) was obtained by Nutraceutica s.r.l. (Monterenzio, Italy). STZ was obtained by Sigma-Aldrich (Milan, Italy).

### 4.3. Establishment of the Diabetic Rat Model

The induction of pancreatic damage was performed by STZ administration. Briefly, a single intravenous injection of STZ at a dose of 60 mg kg^−1^ body weight in freshly prepared citrate buffer was administered on day 1 and glucose blood levels were measured 3 days after STZ administration.

### 4.4. Study Design

Rats were randomly divided into six groups. Each group included 10 animals.

Two weeks later the induction of pancreatic damage, diabetic rats received a daily per os treatment for 4 weeks with the vegetal products suspended in a 1% carboxymethylcellulose (CMC) solution. The control animals were administered with the vehicle only.

Three different groups of diabetic rats were treated as follows: rutin (95%) 25 mg/kg p.o., *M. oleifera* extract (saponins 20%) 50 mg/kg p.o. and *P. ginseng* extract (ginsenosides 10%) 150 mg/kg p.o. A fourth group was treated with the natural product combination: rutin (25 mg/kg), *M. oleifera* extract (50 mg/kg), *P. ginseng* extract (150 mg/kg) p.o. Two further groups, one including non-diabetic rats and the other one untreated diabetic rats, were used as controls.

The choice of doses of the study natural products was based on the availability of previously published data obtained with rutin [44,54], with *M. oleifera* [38,39] and with *P. ginseng* [55,56,57] in rats. In particular, rutin has usually been tested at doses ranging from 25 to 100 mg kg^−1^ [44,54], *M. oleifera* from 10 to 250 mg kg^−1^ [38,39] and *P. ginseng* from 100 to 200 mg kg^−1^ [55,56,57]. In the present study, the criterium applied in the dose selection, also in consideration of the need to administer not only the single compounds but also their combination, was led by the rational choice of low–medium doses found to be efficacious as a single agent in erectile dysfunction rat models. On this basis, a low dose for rutin (i.e., 25 mg kg^−1^), mainly in relation to its hypotensive properties [58], a low–medium dose for *M. oleifera* (50 mg kg^−1^) and a medium dose for *P. ginseng* (i.e., 150 mg kg^−1^) were selected.

### 4.5. Glucose Measurement

Blood glucose values were measured after 4 weeks of treatment by blood sampling from the caudal vein and the analysis was performed with the Accu-Check Aviva planar sensor based on the glucose oxidase method.

### 4.6. Efficacy Evaluation

The efficacy of the study natural compounds as single agents and in combination was evaluated by analyzing blood glucose levels and sexual-related parameters. In particular, the following parameters were evaluated: mount and intromission latencies (as times before the first mount or intromission after the introduction of the female in the cage), the mount and intromission frequencies (as the numbers of mounts or intromission before the first ejaculation) and the ejaculation latency (as the time necessary for the first ejaculation after the introduction of the female in the cage).

### 4.7. Statistical Analysis

All experimental results were expressed as mean ± standard error (M ± SEM). A one-way analysis of variance (one-way ANOVA) was conducted, followed by the Bonferroni test to verify the significance between two averages. The analysis of variance and the Bonferroni test were performed with the statistical program Origin 9.1. Differences with a *p* value < 0.05 were considered significant.

## Figures and Tables

**Table 1:** Glucose blood levels (mg/dL).

Control	STZ	STZ + Rutin	STZ + *M. oleifera* Extract	STZ + *P. ginseng* Extract	STZ + Mixture
108 ± 6.2	364.8 ± 23.5 **	375.3 ± 17.9	289.6 ± 26.2 ^	311.9 ± 15.9 ^	271.2 ± 28.4 ^

Results are reported as mean ± S.E.M. of 10 rats. ** *p* < 0.01 vs. control; ^ *p* < 0.05 vs. STZ.

**Table 2:** Mount latency (seconds).

Control	STZ	STZ + Rutin	STZ + *M. oleifera* Extract	STZ + *P. ginseng* Extract	STZ + Mixture
101.3 ± 5.6	259.6 ± 14.5 **	241.4 ± 16.8	244.7 ± 12.6	263.2 ± 10.3	151.6 ± 8.4 ^^

Results are reported as mean ± S.E.M. of 10 rats. ** *p* < 0.01 vs. control; ^^ *p* < 0.01 vs. STZ.

**Table 3:** Intromission latency (seconds).

Control	STZ	STZ + Rutin	STZ + *M. oleifera* Extract	STZ + *P. ginseng* Extract	STZ + Mixture
108.6 ± 3.3	271.1 ± 18.5 **	243.6 ± 10.1	260.0 ± 8.8	268.8 ± 12.6	160.4 ± 10.1 ^^

Results are reported as mean ± S.E.M. of 10 rats. ** *p* < 0.01 vs. control; ^^ *p* < 0.01 vs. STZ.

**Table 4:** Mount frequency (number).

Control	STZ	STZ + Rutin	STZ + *M. oleifera* Extract	STZ + *P. ginseng* Extract	STZ + Mixture
7.2 ± 0.2	2.2 ± 0.1 **	5.8 ± 0.3 ^	3.4 ± 0.5	5.6 ± 0.2 ^	6.4 ± 0.4 ^^

Results are reported as mean ± S.E.M. of 10 rats. ** *p* < 0.01 vs. control; ^ *p* < 0.05 and ^^ *p* < 0.01 vs. STZ.

**Table 5:** Intromission frequency (number).

Control	STZ	STZ + Rutin	STZ + *M. oleifera* Extract	STZ + *P. ginseng* Extract	STZ + Mixture
17.5 ± 3.1	6.2 ± 1.1 **	6.5 ± 0.8	8.2 ± 2.3	8.4 ± 2.6	13.8 ± 3.6 ^^

Results are reported as mean ± S.E.M. of 10 rats. ** *p* < 0.01 vs. control; ^^ *p* < 0.01 vs. STZ.

**Table 6:** Ejaculation latency (seconds).

Control	STZ	STZ + Rutin	STZ + *M. oleifera* Extract	STZ + *P. ginseng* Extract	STZ + Mixture
345.8 ± 15.6	623.4 ± 22.6 **	636.8 ± 32.1	601.8 ± 18.9	630.5 ± 29.4	552.1 ± 19.7 ^^

Results are reported as mean ± S.E.M. of 10 rats. ** *p* < 0.01 vs. control; ^^ *p* < 0.01 vs. STZ.

## Data Availability

The data presented in this study are available on request from the corresponding author.
